# E2F and p53 Induce Apoptosis Independently during *Drosophila* Development but Intersect in the Context of DNA Damage

**DOI:** 10.1371/journal.pgen.1000153

**Published:** 2008-08-08

**Authors:** Nam-Sung Moon, Luisa Di Stefano, Erick J. Morris, Reena Patel, Kristin White, Nicholas J. Dyson

**Affiliations:** 1 Massachusetts General Hospital Cancer Research Center, Charlestown, Massachusetts, United States of America; 2 Harvard Medical School, Boston, Massachusetts, United States of America; 3 Cutaneous Biology Research Center, Massachusetts General Hospital, Charlestown, Massachusetts, United States of America; Harvard Medical School, Howard Hughes Medical Institute, United States of America

## Abstract

In mammalian cells, RB/E2F and p53 are intimately connected, and crosstalk between these pathways is critical for the induction of cell cycle arrest or cell death in response to cellular stresses. Here we have investigated the genetic interactions between RBF/E2F and p53 pathways during *Drosophila* development. Unexpectedly, we find that the pro-apoptotic activities of E2F and p53 are independent of one another when examined in the context of *Drosophila* development: apoptosis induced by the deregulation of dE2F1, or by the overexpression of dE2F1, is unaffected by the elimination of dp53; conversely, dp53-induced phenotypes are unaffected by the elimination of dE2F activity. However, dE2F and dp53 converge in the context of a DNA damage response. Both dE2F1/dDP and dp53 are required for DNA damage-induced cell death, and the analysis of *rbf1* mutant eye discs indicates that dE2F1/dDP and dp53 cooperatively promote cell death in irradiated discs. In this context, the further deregulation in the expression of pro-apoptotic genes generates an additional sensitivity to apoptosis that requires both dE2F/dDP and dp53 activity. This sensitivity differs from DNA damage-induced apoptosis in wild-type discs (and from dE2F/dDP-induced apoptosis in un-irradiated *rbf1* mutant eye discs) by being dependent on both *hid* and *reaper*. These results show that pro-apoptotic activities of dE2F1 and dp53 are surprisingly separable: *dp53* is required for dE2F-dependent apoptosis in the response to DNA damage, but it is not required for dE2F-dependent apoptosis caused simply by the inactivation of *rbf1*.

**Authors Summary**

E2F1 and p53 are both activated in response to DNA damage and promote the expression of pro-apoptotic genes. In mammalian cells, deregulated E2F1 triggers p53-dependent cell death, and this genetic interaction is thought to explain why lesions in the RB pathway and lesions in the p53 pathway commonly occur in tumors. This study shows that, although E2F and p53 are well conserved in *Drosophila*, the functional connection between the *Drosophila* E2F1 (*de2f1*) and *Drosophila* p53 (*dp53*) genes occurs only when animals are exposed to DNA damage. These results suggest that the basic connection between E2F and p53 stems from their synergistic effects in the DNA damage response, and that the pathways that allow deregulated E2F1 to trigger p53-dependent apoptosis in mammalian cells may be a more recent addition.

## Introduction

Research in mammalian cells has demonstrated that the cellular effects of deregulated E2F activity are intimately connected with the p53 pathway. The p53 pathway provides a surveillance mechanism that is activated in response to a variety of cellular stresses, including DNA damage or oncogene stress. Multiple connections between p53 and RB/E2F are thought to explain why mutations in the pRB pathway (which deregulate E2F and facilitate cell proliferation) and mutations in the p53 pathway (which suppress E2F1-induced apoptosis) strongly synergize in tumorigenesis [1],[2].

Experiments using immortalized cell lines first revealed that elevated levels of E2F1 are sufficient to drive cells from quiescence into S-phase [3]. Cells that are forced to cycle in this manner often undergo apoptosis and, in many cell types, this E2F1-induced apoptosis is p53 dependent [4],[5]. Studies with primary cell cultures revealed that the mutation of p53 not only affects E2F1-induced apoptosis but also facilitates E2F1-driven cell cycle entry by lowering the levels of the Cdk inhibitor p21 [6]. E2F1 can also trigger p53-independent apoptosis and the pro-apoptotic activity of E2F1 has been found to be suppressed by mutation of the p53 related gene p73, or Apaf1, or by inhibition of certain BH3 only proteins [7],[8],[9],[10],[11],[12],[13].

E2F1 and p53 are both activated in response to DNA damage and promote the expression of pro-apoptotic genes [14],[15],[16],[17],[18]. Activation of E2F1, either through the deregulation of pRB or following DNA damage, stabilizes p53 and enhances p53-mediated transcription. E2F1 stabilizes p53 by inducing the expression of p19(p14)/ARF, an inhibitor of the mdm2 ubiquitin ligase that targets p53 for proteolysis [19]. In addition E2F1 also stabilizes p53 via p19(p14)/ARF-independent mechanisms that may include direct binding to p53 and/or changes that promote the phosphorylation of p53 [20],[21],[22]. This activity of E2F, which acts upstream of p53, is thought to be a critical tumor suppressor pathway [23].

The *Drosophila* homologs of p53 and E2F1 have activities that are reminiscent of their mammalian counterparts. dp53 and dE2F1 induce apoptosis when overexpressed and studies of mutant animals show that *dp53* and *de2f1* contribute to DNA damage induced apoptosis [24],[25],[26]. dp53 induces the expression of several pro-apoptotic genes including *reaper*, an inhibitor of DIAP1. Similarly dE2F1/dDP has multiple transcriptional targets, including *hid*, a gene with multiple pro-apoptotic activities.

We, and others, have previously characterized multiple contexts during *Drosophila* development in which deregulated dE2F1 causes apoptosis [27],[28],[29],[30]. Given that E2F and p53 genes are conserved between flies and humans, and the evidence that both dE2F1 and dp53 are important regulators of apoptosis, we anticipated that the strong genetic interactions that exist between p53 and E2F in mammalian cells would also be present in *Drosophila*. Surprisingly, we find that that the pro-apoptotic activities of dp53 and dE2F1 are largely independent of one another during animal development. However, we do find that the activities of these two critical regulators of apoptosis intersect in the context of DNA damage. Our finding suggests that the intimate relationship between p53 and E2F in mammals may have originated from their ability to cooperatively regulate DNA damage-induced cell death.

## Materials and Methods

### *Drosophila* Strains

Unless otherwise noted, all fly crosses were done at 25°C and phenotypes are depicted from female progeny. The *dp53* allele *dp53^−4^* was a generous gift from Dr. Michael Brodsky [31]. The *GUS-dp53* dominant-negative allele (R259H) was obtained from the Bloomington Stock Center. The following stocks were previously published in Morris et al, 2006: *GMR-GAL4,UAS-dp53/CyO ftz lacZ; GMR-GAL4,UAS-dE2F1, UAS-dDP/CyO ftz lacZ; Act88F-GAL4,UAS-dE2F1/CyO ftz lacZ; nos-GAL4,UAS-dE2F1/CyO ftz lacZ; Sca-GAL4,UAS-dE2F1/CyO ftz lacZ*. The following stocks were previously published as indicated: *dDP* mutants: *dDP^a4^/dDP^a2^* [32], *hid* mutants: *hid^05014^/Df(3L)X14, rbf1* mutants: *rbf1^120a^* and *rbf1^Δ14;^FRT19A* was used to generate clones in eye discs by crossing with *y,w,GFP^ubi^,FRT19A;ey-FLP* [19]. The *reaper* mutant was generated by imprecise excision of *P{SUPor-P}KG07184* [33]. The deletion of the genomic region was confirmed by sequencing and determined to extend from 1860 bp 5′ of the *reaper* transcript to 661 bp downstream.

### Irradiation of Larvae

Third instar larvae were exposed to 40 Gy of gamma-ray using cesium irradiator. For each experiment, minimum of ten larvae were used for analysis. Representative images are shown in the Figures.

### Immunocytochemstry and *in situ* Hybridization

To visualize apoptotic cells rabbit poly clonal antibodies raised against the cleaved form of humans Caspase 3, anti-C3 antibodies (Cell Signaling), were used with 1∶100 dilution. For immunostaining, discs were fixed with 4% formaldehyde for 30 minutes at 25°C, washed twice with 0.3% PBST (0.3% Triton X-100 in PBS) for 5 minutes at room temperature, then incubated with desired primary antibody in 0.1% PBST (0.1% Triton X-100 in PBS) with 5% normal goat serum at 4°C for 16 hours. After washing five times with 0.1% PBST for 10 minutes, at room temperature, discs were incubated with secondary antibody in 0.3% PBST (0.3% triton in PBS) with 5% normal goat serum at 4°C for 16 hours. After washing five times with 0.1% PBST for 10 minutes, at room temperature, wing discs were mounted for confocal microscopic imaging.

### Image Quantification

ImageJ software [34] was used to measure the fluorescence intensity (mean gray values) of confocal images. Images were analyzed from at least five disc images taken from the same sets of experiments.

### Real Time qPCR

Total RNA was prepared from eye-antenna discs with Trizol (Invitrogen) reagent and reverse transcription PCR (RT-PCR) was performed using Taq Man Reverse Transcription (PE Applied Biosystems) according to manufacture specification. Real time PCR was performed using an ABI prism 7700 Sequence Detection system. Relative levels of specific mRNAs were determined using the SYBR Green I detection chemistry system (Applied Biosystems Foster City, CA). Quantification was performed using the comparative C_T_ method as described in the manufacturer procedures manual. *Rp49* was used as normalization control. All primers were designed with Primer Express 1.0 software (Applied Biosystems Foster City, CA) following the manufacturer's suggested conditions. The primer pairs used were:

RP49-58F TACAGGCCCAAGATCGTGAAG

RP49-175R GACGCACTCTGTTGTCGATACC

Hid-1095F CATCAGTCAGCAGCGACAGG

Hid-1196R ACGAAAACGGTCACAACAGTTG

RNR21180F CATCTGCCAGATGTCGTGGTAC

RNR21282R GAAGTCCGTAACCCCCTTCG

Reaper-128F CCAGTTGTGTAATTCCGAACGA

Reaper-241R GGATCTGCTGCTCCTTCTGC

## Results

In previous studies we exploited the *GAL4/UAS*-system to examine the effects of ectopic dE2F1 expression during *Drosophila* development [29]. Elevated dE2F1 activity causes inappropriate cell proliferation and/or apoptosis and the balance between these outcomes varies in different developmental contexts. Using tissue specific drivers we have generated stable stocks with visible phenotypes that are caused by dE2F1-induced apoptosis. One such stock, *Act88F-GAL4, UASdE2f1*, has multiple wing defects that can be fully suppressed by the co-expression of RBF1 or by the expression of the caspase inhibitor p35. Previously we used *Act88F-GAL4, UASdE2f1* to find novel modifiers of dE2F1-induced apoptosis that are conserved in mammalian cells [29].

**Figure 1:**
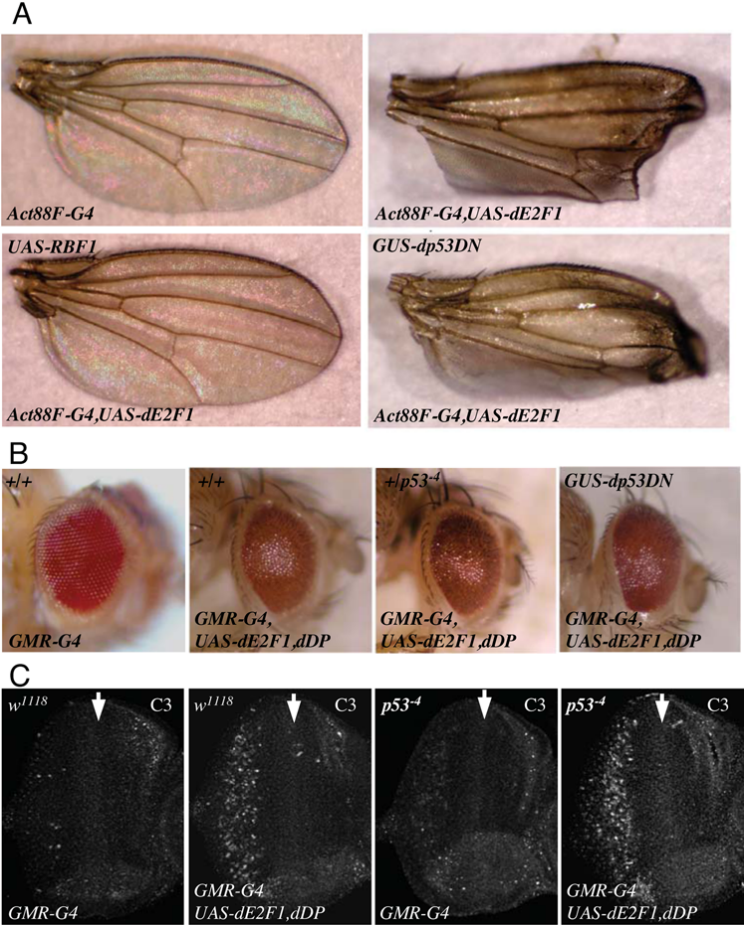
dp53 is not required for dE2F1-induced cell death. (A) The wing phenotype of *Act88F-Gal4,UAS-dE2F1* was suppressed by co-expression of RBF1 but was unaffected by co-expression of dp53DN. *GUS* is a *GMR* vector derivative that contains binding sites for GAL4 proteins [34]. (B) The rough eye phenotype of *GMR-Gal4; UAS-dE2F1,UAS-dDP* flies was unaffected by co-expression of dominant negative p53 (dp53DN) or by a mutant allele of p53. The *Drosophila* wing and eye phenotypes shown in this figure are representative figures of at least 50 flies per genotype scored. No significant variation in the phenotypes was observed. (C) Apoptotic cells were visualized by C3 (an antibody that recognizes the active form of Caspase 3) staining of third instar eye discs of the genotypes shown. *GMR-Gal4* drives the expression dE2F1 and dDP in the posterior region of the eye disc inducing a pattern of apoptosis that was unaffected by the homozygous mutation of *dp53*. (Hereon, the white arrow indicates the position of the morphogenetic furrow)

In mammalian cells, E2F-induced apoptosis can occur via p53-dependent and p53-independent pathways. We used genetic tools described above to investigate the role of p53 in E2F-induced apoptosis in *Drosophila*. Strikingly, we found that the *Act88F-GAL4, UASdE2f1* wing phenotype was completely unaffected by mutant alleles of *dp53* or by co-expression of a dominant negative form of dp53 ([29] and Figure 1A). To exclude the possibility that this unexpected result was an unusual feature of this particular phenotype we performed similar experiments using phenotypes induced by ectopic dE2F1 in either the developing eye or bristles. These phenotypes were also unaffected by mutant alleles of *dp53* or by expression of dp53DN (Figure 1B and data not shown), indicating that the lack of an interaction between *dp53* and *dE2F1* is a reproducible result that is true in several different genetic assays.

The adult phenotypes of these dE2F1-expressing stocks provide only an indirect measure of dE2F1-induced apoptosis. It was conceivable that the inactivation of dp53 might alter the level or distribution of dE2F1-induced apoptosis without changing the eventual visible phenotype of the stocks. To test this, we used C3, an antibody that recognizes activated caspase, to directly monitor the appearance of apoptotic cells in eye imaginal discs expressing dE2F1/dDP, in either the presence or absence of endogenous dp53. *GMR-GAL4* driven expression of *UAS-dE2F1* and *UAS-dDP* strongly induced apoptosis in eye discs that are wild-type for *dp53*. However, no appreciable difference in the level or pattern of C3 staining was observed following dE2F1/dDP expression in wild-type or *dp53* mutant eye discs (Figure 1C).

**Figure 2:**
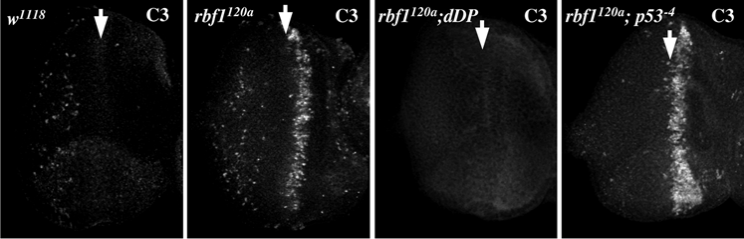
Mutation of *dp53* has no effect on dE2F1/dDP-dependent cell death in *rbf1* mutant eye discs. Third instar eye discs of the indicated genotypes were stained with C3 to visualize apoptotic cells. A wave of cell death in *rbf1^120a^* mutant eye discs was eliminated by the homozygous mutation of *dDP* but not by the homozygous mutation of *dp53*.

To test the possibility that this lack of interaction was an artifact of dE2F1 overexpression, we examined a wave of apoptosis that occurs in eye discs that are hypomorphic for *rbf1*. RBF1 is a key regulator of dE2F1 and *rbf1^120^* mutant flies have elevated dE2F1 activity. Previous studies have shown this wave of apoptosis, that accompanies the morphogenetic furrow as it crosses the eye discs during the third larval instar, is caused by deregulated E2F activity and can be eliminated by mutation of either *de2f1* or *dDP* [28]. dDP is an essential heterodimeric partner for both *Drosophila* dE2F proteins but unlike *de2f1* mutants, that are not embryonic lethal, *dDP* mutants develop to pupal stages. *dDP* mutants thus provide a simple way to examine imaginal discs that lack any E2F/DP complexes. Whereas the wave of apoptosis in *rbf1* mutant eye discs was completely suppressed by mutation of *dDP*, it was unaffected by mutation of *dp53* (Figure 2). Hence, this wave of apoptosis that is triggered by the release of endogenous dE2F1/dDP from RBF1 is independent of *dp53*.

**Figure 3:**
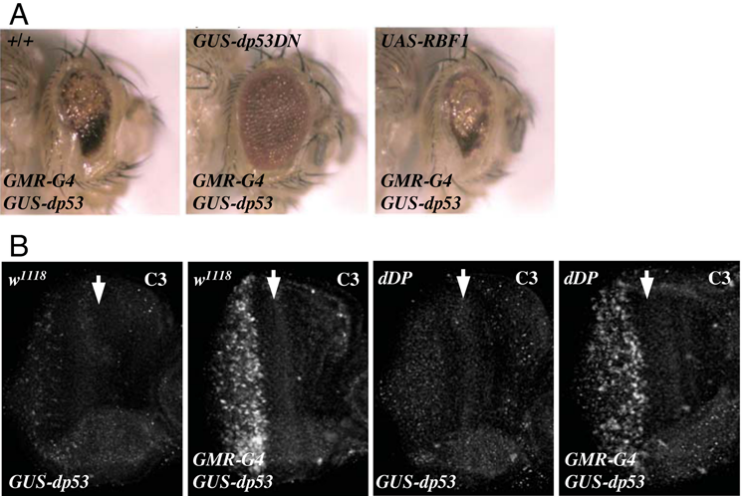
dDP is dispensable for dp53- induced cell death. (A) A rough eye phenotype was generated using *GMR-Gal4* to drive expression of *dp53*. The dp53-dependent eye phenotype was suppressed by co-expression of dominant negative dp53 but was unaffected by co-expression of RBF1. The eye phenotypes shown in this figure are representative figures of at least 50 flies per genotype scored (B) dp53-induced apoptosis was visualized in third instar eye discs by immunostaining with C3. The *GUS-dp53* transgene did not induce cell death by itself, but *GMR-Gal4* driven expression of *dp53* caused widespread apoptosis at the posterior part of the eye discs. dp53-induced cell death was unaffected by homozygous mutation of *dDP*.

To be thorough, we also examined the converse possibility that dp53-induced apoptosis might require E2F activity. *GMR-GAL4* driven expression of *dp53* induces apoptosis in the developing eye disc, giving a rough eye phenotype that can be suppressed by co-expression of dominant negative form of dp53. This phenotype was unaffected by the co-expression of RBF1 (Figure 3A), which suppresses dE2F1 activity. Moreover, when we compared the patterns of dp53-induced apoptosis in wild-type eye discs and in eye discs mutant for *dDP*, we found that *GMR-GAL4* driven expression of dp53 efficiently induced apoptosis in eye discs of control larvae and showed no detectable change in *dDP* mutant discs. Hence, the pro-apoptotic activity of *dp53* does not require functional E2F/DP complexes (Figure 3B).

Together these results show that, while elevated levels of dp53 and dE2F1 can both induce apoptosis, these activities cannot be placed in a linear genetic pathway with one either upstream or downstream of the other.

**Figure 4:**
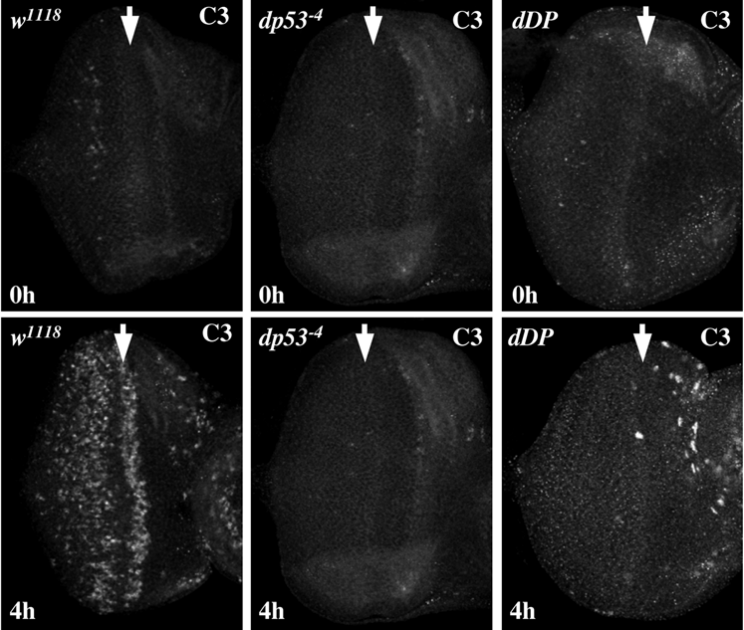
DNA damage-induced cell death in eye discs is suppressed by the mutation of either *dDP* or *dp53*. Third instar larvae of indicated genotypes were treated with 40 Gy of ionizing irradiation. C3 immunostaining was used to detect apoptotic cells in eye imaginal discs either before (0 h) or 4 h after (4 h) irradiation.

Next we considered the possibility that the dE2F1-dependent apoptosis that occurs during animal development might have different requirements to the dE2F1-dependent apoptosis that occurs in response to DNA damage. There are few apoptotic cells in wild type *Drosophila* third instar eye discs. However, a distinct pattern of cell death occurs within four hours of irradiation (Figure 4). Cells in the Morphogenetic Furrow (MF) are protected from DNA damage-induced apoptosis while cells near the MF are sensitive to DNA damage-induced cell death. The DNA damage-induced cell death seen in irradiated eye discs requires the activities of both dp53 and dE2F1 (Figure 4). Virtually no apoptotic cells were observed in *dp53* mutant eye discs after treatment with ionizing irradiation. A similar result was observed in *dDP* mutant eye discs that lack functional *de2f1*. The fact that dE2F/dDP and dp53 are both required for DNA damage-induced cell death shows that, in this context, neither transcription factor is sufficient to induce apoptosis in the absence of the other. Because of this, we used irradiated eye discs as a setting to look for a functional interaction between dp53 and the dE2F1/dDP activity that is deregulated in *rbf1* mutant cells.

**Figure 5:**
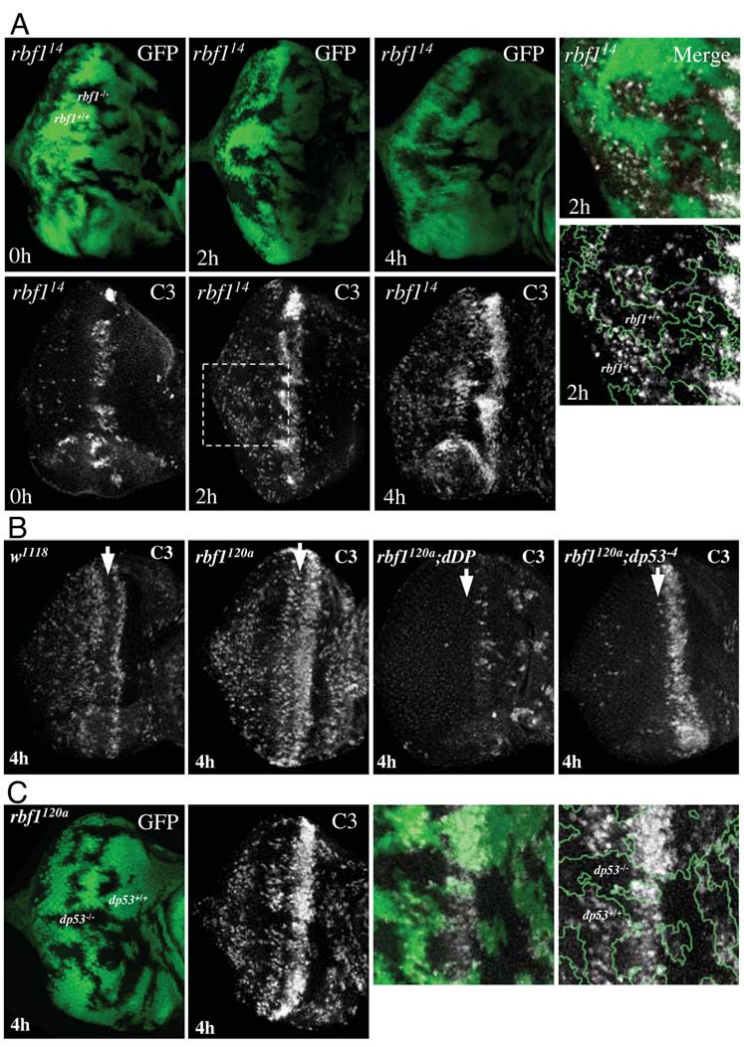
rbf1 suppresses DNA damage-induced apoptosis, and the elevated apoptosis seen in *rbf1* mutant discs requires both *dDP* and *dp53*. (A) *rbf1* mutant cells show a heightened sensitivity to DNA damage-induced cell death. Somatic clones of *rbf1^14^* null mutant cells were generated in third instar larvae using *ey-FLP*. Discs were treated with 40 Gy of ionizing irradiation. Apoptotic cells in the eye discs before (0 h) and 2 h and 4 h after irradiation (2 h and 4 h) were detected by C3 staining. The right panel is a magnified view of the eye discs 2 h after irradiation. The clonal boundary is indicated by a green line. (B) Third instar larvae of indicated genotypes were treated with 40 Gy of ionizing irradiation and apoptotic cells were visualized with C3 4 h later. Unirradiated discs of the same genotypes are shown in Figure 2. Note that the elevated level of apoptosis that occurs throughout irradiated *rbf1* mutant discs is p53-dependent, while the stripe of apoptosis in *rbf1* mutant eye discs that appears in the MF during development and occurs in irradiated and non-irradiated discs is p53-independent. Mutation of *dDP* suppresses both patterns of apoptosis in irradiated *rbf1* mutant discs. (C) Somatic clones of *dp53^−4^* mutant cells were generated in third instar larvae of *rbf1^120a^* using *ey-FLP*. The Discs were treated with 40 Gy of ionizing irradiation, apoptotic cells in the eye discs 4 h after irradiation were visualized by C3 staining. The right panel shows that a magnified view of the morphogenetic furrow region (MF) of the discs and the green line marks the clone boundary.

As a first step, we examined the pattern of DNA-damage-induced apoptosis in *rbf1* mutant cells. Using *ey-FLP* and *FRT-rbf1^14^* we generated mosaic eye discs carrying somatic mutant clones that were null for *rbf1*. This allowed a side-by-side comparison between wild-type cells and mutant cells with deregulated dE2F1. As described earlier, *rbf1* mutant cells are prone to apoptosis in the MF. When the mosaic discs were irradiated, *rbf1^14^* mutant cells show a heightened sensitivity to apoptosis (Figure 5A). More C3 staining was detected in *rbf1* mutant clones after irradiation, and this staining appears more quickly in the mutant cells. A similarly elevated pattern of DNA-damage induced apoptosis was also seen in hypomorphic *rbf1* mutant eye discs, *rbf1^120^* (Figure 5B). Here the increase in apoptosis was evident in a broadening of the stripe of apoptosis at the MF, in the appearance of apoptotic cells in the anterior region of this disc containing proliferating cells, and in the appearance of apoptotic cells among the post-mitotic cells posterior to the MF. The intensity of the C3 staining was measured using image software. This quantification shows a 2.1 fold increase in staining at the posterior regions of *rbf1^120^* mutant eye discs, and a 2.6 fold increase in the anterior regions of the discs.

Next we asked whether this heightened sensitivity of *rbf1* mutant cells to apoptosis requires p53 or E2F (Figure 5B). The elevated level of DNA-damage-induced apoptosis in *rbf1^120a^* mutant eye discs was suppressed by mutation of *dDP*, consistent with the idea that this change is caused by deregulated dE2F1. In addition, this elevated level of apoptosis was also eliminated by the mutation of *dp53*, indicating that this dE2F1-dependent apoptosis is also dependent on dp53. Remarkably, within the same disc, the wave of dE2F1/dDP-dependent apoptosis in the MF, that appears in the absence of irradiation (Figure 2), was unaffected by the mutation of dp53 (Figure 5B). These results show that irradiated *rbf1* mutant eye discs contain two distinct patterns of apoptosis: a developmentally induced wave of apoptosis that is dE2F1/dDP dependent but independent of dp53, and a hypersensitivity to DNA-damage induced apoptosis that depends on both dE2F1/dDP and dp53. Hence, dp53 is not required for E2F-dependent apoptosis that occurs during animal development following the mutation of *rbf1,* but it is required for dE2F-dependent apoptosis in the context of a DNA damage response. This includes the broadening of the stripe of apoptosis at the MF (Figure 5C).

How do deregulated dE2F1 and dp53 cooperate to sensitize cells to apoptosis following DNA damage? Potentially, dE2F1 and dp53 might regulate different pro-apoptotic genes and these targets could collectively promote cell death. Alternatively, dE2F1 and dp53 might converge on a common target that is critical for DNA damage-induced cell death. Previous studies have suggested that the pro-apoptotic genes *hid* and reaper are likely to be points of convergence between dp53 and dE2F1. Both genes are up-regulated following DNA damage in a *dp53*-dependent manner and both have also been identified as dE2F1-regulated genes [24],[25],[27],[35].

**Figure 6:**
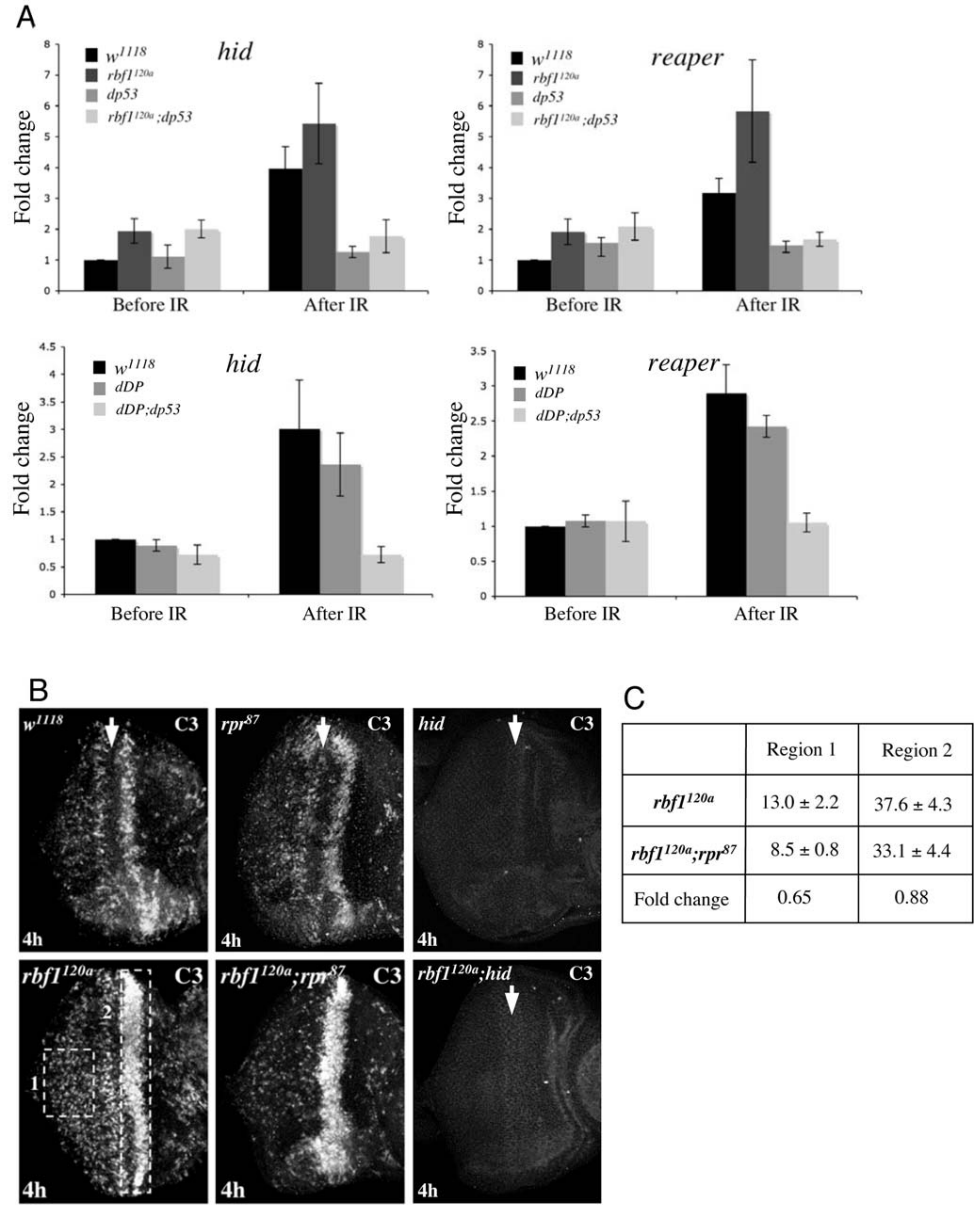
*hid* and *reaper* are deregulated, and functionally important, for DNA damage-induced apoptosis in *rbf1* mutant eye discs. (A) Eye discs from control and *rbf1* mutant third instar larvae were dissected before and three hours after ionizing irradiation. RNA samples were isolated from each genotype and the expression levels of indicated genes were measured by quantitative real-time PCR. Relative expression levels are shown from three independent experiments, each with triplicate samples. A similar analysis was performed using *dDP* and *dDP;dp53* mutant larvae. (B) Effects of *reaper* and *hid* mutation in *rbf1* mutant eye discs are shown. 4 h after irradiation treatment, apoptotic cells in the indicated eye discs were visualized by C3. *hid* Mutation completely suppresses DNA damage-induced cell death throughout both control and *rbf1* mutant eye discs. (C) The intensity of C3 staining signals in *rbf1* mutant and *rbf1;rpr* mutant eye discs was quantified using the Image J software. The fold change indicates that the *reaper* mutation results in a more significant reduction of the level of DNA damage-induced cell death posterior to the MF (region 1 indicated in B) compared to the MF region (region 2 indicated in B) in *rbf1* mutant discs.

Using quantitative PCR, we measured the level of *hid* and *reaper* mRNA, both before and after DNA damage, in control, *rbf1*, *dp53*, and *rbf1;dp53* mutant eye discs (Figure 6A). In control discs, DNA damage increased the levels of *hid* and *reaper* mRNAs 4.0 and 3.2 fold respectively. Both genes were also expressed at an elevated level in *rbf1* mutant eye discs prior to DNA damage, again with a similar increase (2.0 fold for *hid*, 1.9 fold for *reaper*). Interestingly, this increase in *rbf1* mutant eye discs is *dp53* independent–since mutation of *dp53* mutation had no effect on *hid* and reaper expression in (unirradiated) *rbf1* mutant animals. Irradiation of *rbf1* mutant eye discs further increased the expression of *reaper* (5.8 fold) compared to the irradiated control eye discs but, interestingly, gave only a very slight increase in the level of *hid* mRNA (5.4 fold) compare to the control. These changes were specific to these pro-apoptotic targets, since expression of *rnr2*, a cell cycle-regulated target of dE2F1, did not show the same increase (data not shown). These results demonstrate that elevated dE2F1 activity (resulting from the inactivation of *rbf1*) and DNA damage-induced activation of dp53 have co-operative effects on the expression of some pro-apoptotic genes.

We measured the level of *hid* and *reaper* mRNA, both before and after DNA damage, in control, *dDP*, and d*DP;dp53* mutant eye discs. The levels of *hid* and *reaper* expression were similar in control and *dDP* mutant eye discs, both before and after irradiation. This indicates that the ability of dp53 to activate *hid* and *reaper* after irradiation does not require dE2F/dDP activity. In summary, and in agreement with the genetic experiments presented in Figure 1 and 3, dE2F1 and dp53 cooperate after DNA damage but their abilities to activate transcription of pro-apoptotic genes do not depend on one another.

To test the functional significance of these gene expression changes we examined the effects of removing either *hid* or *reaper* on the level of DNA damage-induced apoptosis in wild-type or *rbf1* mutant eye discs. Previous studies have shown that the gene dosage of *hid* affects the levels of DNA damage-induced cell death [35] but it has not been possible to compare the effects of homozygous mutation of *reaper* and *hid*, in large part because of the lack of specific mutant alleles of *reaper*. For these experiments we used a recently generated, specific allele of *reaper*, *rpr^87^*, and a combination of viable *hid* mutant alleles (see materials and methods).

Strikingly, mutation of *hid* completely abolished DNA damage-induced apoptosis in control and *rbf1* mutant discs (Figure 6B). In contrast, mutation of *reaper* had different consequences in control and *rbf1* mutant discs. Mutation of *reaper* caused no appreciable change in the level of DNA damage-induced cell death in the presence of RBF1, however, the level of cell death seen in *rbf1^120a^* discs was clearly reduced when *reaper* was mutated (Figure 6B). Interestingly, measurement of C3 staining intensity showed that the *reaper* mutation had greater effects at the posterior region of the MF while only a slight change was evident in the vicinity of the MF (Figure 6C). It is important to point out that *reaper* mutations did not affect the level or pattern of cell death in *rbf1^120a^* eye discs before irradiation treatment (data not shown). In summary, we conclude that the expression of *hid* is very important for the apoptosis induced by DNA-damage in wild-type discs. Similarly, the wave of apoptosis that occurs in *rbf1* mutant discs requires *hid* but not *reaper*. However, irradiation of *rbf1* mutant discs causes a further increase in *reaper* expression and a more extensive pattern of apoptosis that is dependent on both *reaper* and *hid*.

## Discussion

In both mammalian cells and in *Drosophila*, an elevated level of E2F1/dE2F1 activity delays cell cycle exit and sensitizes cells to apoptosis. Studies in mammalian cells have led to the conclusion that, in many cellular contexts, E2F1 acts upstream of p53. In the experiments described above we show that while homologs of p53 and E2F1 exist in *Drosophila,* the functional relationship between dp53 and dE2F1 is different from that seen in mammalian cells. Epistasis experiments demonstrate that *de2f1*-induced apoptosis does not require *dp53*, and that *dp53*-induced apoptosis does not require dE2F activity. Consequently, animal phenotypes caused by the over-expression of *de2f1* and attributable to *de2f1*-induced apoptosis are unaffected by mutation of *dp53*. However, we do find that, like in mammals, dE2F1 can cooperate with dp53 to promote DNA damage-induced cell death.

**Figure 7:**
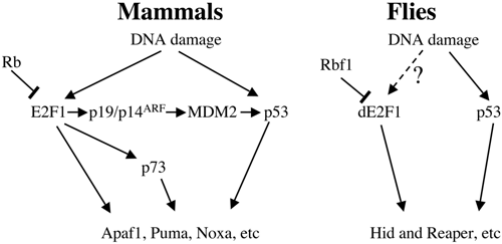
E2F and p53 network between mammals and flies. In mammalian cells, the activation of E2F1 is sufficient to activate p53. One of the key links between E2F and p53, is provided by p19/p14^ARF^ and mdm2. *Drosophila* lack any clear orthologs of p19/p14^ARF^ and mdm2 and lack of this connection may limit the crosstalk between E2F1 and p53. In the context of DNA damage, where dE2F1 and dp53 converge on a common set of pro-apoptotic target genes, dE2F1 and dp53 co-operate to promote cell death. While DNA damage is shown to activate E2F1 and p53 pathways in mammals, it remains uncertain whether dE2F1 is similarly activated by DNA damage.

It is satisfying when homologous genes have conserved functions, but it is perhaps more intriguing when they do not. Why are the genetic interactions between E2F1/dE2F1 and p53/dp53 so different between flies and humans? One general explanation is that *de2f1* and *dp53* are unique genes in *Drosophila*, whereas *E2f1* and *p53* are members of families of related mammalian genes. p53, p63 and p73 have distinct functions, as do E2F1, E2F2 and E2F3, and the roles of dp53 and dE2F1 may resemble the function of an ancestral protein, rather than the more specialized roles of the individual mammalian family members. A second, and more detailed explanation, is that a key connection between E2F1 and p53 is provided in mammalian cells by p19 (p14)/ARF and Mdm2. The *Drosophila* genome lacks clear homologs of either of these genes. Hence, a major pathway by which E2F1 induces p53 in mammalian cells is likely to be absent in *Drosophila* (Figure 7). Although it is possible that functional homologues might exist in *Drosophila*, the absence of genetic interaction presented in this study argues otherwise. Interestingly, *dp53* also fails to induce expression of *dacapo* (the *Drosophila* ortholog of p21^CIP1^) and also appears to be defective for G1/S checkpoint functions [26],[35]. *Drosophila* cells arrest primarily at G2/M in response to DNA damage, and there is scant evidence for G1/S damage checkpoint in irradiated discs. Thus, while *dp53* is important for DNA-damage induced apoptosis, its activity appears to be largely uncoupled from some of the key sensors and effectors of mammalian p53 checkpoint pathways.

As seen in mammals, the expression of critical pro-apoptotic genes is regulated by both E2F and p53 in *Drosophila*. The convergence of dE2F1 and dp53 on common target genes provides a simple molecular explanation for how dE2F1 and dp53 cooperate to promote DNA damage-induced cell death (Figure 7). Our result indicates that elevated dE2F1 activity can influence the basal level of *hid* and *reaper* expression while dp53 is essential for the induction of gene expression after DNA damage (Figure 6A). This difference in the physiological context in which they function may explain why dE2F1 and dp53 proteins act independently of one another and appear to function in parallel pathways. Interestingly, the levels of *hid* and *reaper* expression before irradiation were unaffected by mutation of *dDP*. This indicates that dE2F1 does not contribute to the basal expression of these pro-apoptotic genes. This is somewhat expected since *hid* and *reaper* are not expressed in a cell cycle-dependent manner and are likely to be expressed at a low level in the absence of stress. Moreover, since *hid* and *reaper* are induced at a similar level between control and dDP mutant eye discs after irradiation, there must be other apoptotic genes regulated by dE2F that are responsible for the insensitivity to DNA damage in *dDP* mutant eye discs. Precisely how dE2F1 and dp53 synergize in transcriptional regulation is unclear. The effects of dE2F1 and dp53 on the transcription of these targets may include both direct effects at the promoter and indirect effects on the organization of the larger H99 locus. Indeed, a recent study identified a region upstream of *reaper* that can influence the expression of *hid* [36].

There are striking parallels between the results described here and studies of *Rb* mutation in the mouse. Tissue specific ablation of *Rb* in the developing retina triggers apoptosis in many cell types. Much of the developmental apoptosis seen in Rb-mutant retina is p53-independent [37],[38]. Curiously however, tumorigenesis in these tissues is limited by the action of p53 and many retinoblastomas select for amplification of a region that includes Mdmx [39]. This distinction is highly reminiscent of the *rbf1* mutant discs shown in Figure 5, where the wave of dE2F1-dependent apoptosis that occurs during eye development is independent of *dp53*, but the hypersensitivity of *rbf1* mutant cells to DNA damage induced apoptosis requires both dE2F and *dp53*. Given the recent studies showing that an activated DNA damage response is a hallmark of pre-neoplastic lesions [40], it is particularly interesting that DNA damage-induced apoptosis is one setting where the strong synergy between E2F and p53 is conserved between flies and humans.
